# Plasma Progranulin Concentrations Are Increased in Patients with Type 2 Diabetes and Obesity and Correlated with Insulin Resistance

**DOI:** 10.1155/2013/360190

**Published:** 2013-02-17

**Authors:** Hua Qu, Huacong Deng, Zhenping Hu

**Affiliations:** Department of Endocrinology, The First Affiliated Hospital of Chongqing Medical University, Chongqing 400016, China

## Abstract

Insulin resistance (IR) is considered to be one of the most important pathogenesis of glycolipid metabolism disorders. However, the molecular mechanism responsible for IR is not fully understood. Recently, the chronic inflammation has been proposed to be involved in the pathogenesis of IR. In this study, we aim to investigate the concentrations of plasma progranulin in Chinese patients with obesity (OB) and type 2 diabetes mellitus (T2DM), and its relationship to IR. Plasma progranulin concentrations were significantly higher in the T2DM patients than in the normal glucose tolerant (NGT) subjects (*P* < 0.01). Within the T2DM and the NGT patients, the concentrations of progranulin were significantly higher in obese subjects than that in the normal weight subjects (225.22 ± 34.39 ng/mL versus 195.59 ± 50.47 ng/mL and 183.79 ± 61.63 ng/mL versus 148.69 ± 55.27 ng/mL, *P* < 0.05). Plasma progranulin concentrations correlated positively with weight, waist circumferences, BMI, HbA1c, TG, IL-6, FINS and HOMA-IR (*P* < 0.05), while correlated negatively with HOMA-**β** (*P* < 0.05). Multiple linear regression analysis showed that BMI, HbA1c, IL-6 and TG correlated independently with circulating progranulin concentrations (*P* < 0.05). These results suggested that Plasma progranulin concentrations were higher in Chinese patients with type 2 diabetes and obesity and correlated closely with glycolipid metabolism, chronic inflammation and IR.

## 1. Introduction

Progranulin, also known as granulin/epithelin precursor (GEP), acrogranin, and PC cell-derived growth factor (PCDGF), is a 593 amino acid growth factor [1, 2]. Progranulin gene can be found in adipose tissue, epithelial tissue, gastrointestinal tract, reproductive organs, and so forth [3]. The widespread expression of PGRN gene makes it act in many physiological and pathophysiological processes, such as the inflammatory response, the injury-induced tissue repair, and the tumor formation progression [3, 4]. Previous studies have demonstrated that increased gene expression of PGRN stimulates cancer cell division, invasion, and against anoikis, promoting tumor formation [2]. Loss-of-function mutations of PGRN gene lead to the onset of some neurodegenerative diseases such as frontotemporal dementia [5, 6]. Recent studies showed that PGRN could restrain rheumatoid arthritis by binding directly to tumor necrosis factor receptors (TNFR) and play an anti-inflammatory role in the processes [7]. It has been reported that circulating PGRN levels are elevated in patients with type 2 diabetes [8]. Moreover, increased plasma PGRN levels are associated with impaired glucose tolerance rather than impaired fasting glucose [9]. Although type 2 diabetes is often accompanied by obesity, the respective role of elevation of circulating PGRN levels in obesity and type 2 diabetes remains to be established. 

Insulin resistance (IR) is a key feature of type 2 diabetes and obesity [10]; as yet the molecular mechanism responsible for IR is not fully understood. Recently, the chronic inflammation has been proposed to be involved in the pathogenesis of IR [11, 12]. Compared to the healthy subjects, plasma levels of the proinflammatory markers are increased (e.g., TNF-*α*, IL-6, and resistin), while the anti-inflammatory markers are decreased (e.g., adiponectin, leptin) in the obese subjects with type 2 diabetes [11, 12]. PGRN has been shown to promote IR in obese mice and 3T3-L1 adipocytes through IL-6. However, the effect of PGRN on IR in obese patients with type 2 diabetes is still not known. Thus, we investigated the changes in plasma PGRN concentrations in Chinese patients with obesity and type 2 diabetes and analyzed the relationship of PGRN with IR.

## 2. Methods

### 2.1. Subjects

Eighty patients with newly diagnosed type 2 diabetes and 88 subjects with normal glucose tolerance (NGT) were recruited in this study, and age of the subjects ranged from 40 to 75 years. 75 g oral glucose tolerance test (OGTT) was performed in all 168 subjects. All of the T2DM patients were newly diagnosed and had not received any antidiabetes treatments including diet, exercise, and medications. The diagnoses of T2DM were based on the diagnostic criteria of World Health Organization (WHO) in 1999, and then according to the WHO-Western Pacific Region diagnostic criteria (2000) [13] defined obesity as BMI ≥25 kg/m^2^. All subjects were divided into four subgroups: NGT-normal weight (NGT-NW) subgroup, NGT-obesity (NGT-OB) subgroup, T2DM-NW subgroup, and T2DM-OB subgroup. 

### 2.2. Exclusion Criteria

1. Smoking and drinking history.
2. Acute and chronic complications of diabetes.
3. Sustained hypertension, coronary heart disease.
4. Acute and chronic inflammatory diseases as determined by clinical symptom of infection, blood leukocyte >7 × 10^9^/L, or high-sensitivity C-reactive protein (hs-CRP) >5.0 mg/dL;
5. Hepatic or renal disease and systemic corticosteroid treatment.
6. Women who were currently pregnant and breastfeeding were also excluded from this study.

The study was approved by the Ethical Committee of Chongqing Medical University. Signed informed consents were obtained from all participants in this study.

### 2.3. Study Measurements

#### 2.3.1. Clinical Evaluation of Subjects

Standardized protocols were used to measure height, body weight, waist circumferences, hip circumferences, and blood pressure (BP) in all subjects. Height, waist, and hip circumferences were measured to minimum recorded unit 0.1 cm, body weight was measured to an accuracy of ±0.2 kg, and blood pressure was measured twice with a standard mercury manometer with the subjects seated and was used for the second measurement. Body mass index (BMI) and waist to hip ratio (WHR) were calculated. 

Overnight fasting blood samples were collected for the determination of fasting plasma glucose (FPG), HbA1c, fasting insulin (FINS), triglyceride (TG), total cholesterol (TC), high-density lipoprotein cholesterol (HDL-c), low-density lipoprotein cholesterol (LDL-c), and liver and kidney functions. Blood samples also were collected after 2 hours of a 75 g OGTT for determined the 2 h plasma glucose (2hPG). All of the blood samples were separated within 1 h and then frozen at −80°C until used in this study, all within 3 month period. Glucose was assayed by glucose oxidase method. HbA1c was measured by isoelectric focusing. Fasting insulin (FINS) was measured in serum by RIA using human insulin as standard (Linco, St Charles, MO, USA). Lipid profiles, liver and kidney functions were detected by biochemical autoanalyzer (Beckman CX-7 Biochemical Autoanalyser, Brea, CA, USA).

#### 2.3.2. Assessment of Plasma Progranulin and IL-6 Concentrations

Plasma PGRN and IL-6 concentrations were determined by enzyme-linked immunosorbent assays according to the manufacturers' instructions. (Human ELISA kit, CUSABIO Science Co, Ltd, China). All samples were run in duplicate and repeated if there was a >15% difference between duplicates. No significant cross-reactivity or interference was observed.

#### 2.3.3. Related Calculation Formulas

Body mass index (BMI) formula is weight in kilograms divided by height in meters squared.

The Homeostasis Model Assessment for insulin resistance (HOMA-IR) was computed as follows: Fasting insulin (mU/L) × Fasting plasma glucose (mmol/L)/22.5.

The Homeostasis Model Assessment for *β*-cell function (HOMA-*β*) was calculated using the following formula: 20 × Fasting insulin (mU/L)/Fasting plasma glucose (mmol/L) − 3.5.

### 2.4. Statistical Analysis

SPSS software, version 19.0 (IBM, Armonk, NY), was used for all statistical analyses. Data are expressed as mean values ± standard deviation (SD). Before statistical analysis, nonnormally distributed parameters were logarithmically transformed to approximate a normal distribution. Independent-samples *t*-tests were used to compare continuous variables between the 2 groups. Analysis of variance and Student-Newman-Keuls tests were performed for multiple and pairwise comparisons, respectively. Interrelationships between variables were analyzed by Pearson correlation analysis. Multiple linear regression analyses were used to determine independent predictors of plasma PGRN. *P* values <0.05 were considered to be statistically significant.

## 3. Results

### 3.1. The Clinical Characteristics

The anthropometric and metabolic parameters in different subgroups were shown in Table 1. Between NGT and T2DM groups, there were no significant differences in age, weight, BMI, waist circumferences, and WHR. Compared to NGT group, the T2DM group exhibited higher levels of SBP, FPG, 2hPG, FINS, HbA1c, TG, LDL-c, IL-6, hs-CRP, and HOMA-IR (*P* < 0.05 or *P* < 0.01). The levels of HDL-c, HOMA-*β* were significantly higher in NGT group than in T2DM group (*P* < 0.01). SBP was higher in T2DM-NW and T2DM-OB subgroups than in NGT-NW subgroup, while DBP was higher in T2DM-OB subgroup than in T2DM-NW subgroup (*P* < 0.05). The levels of BMI, waist circumferences, FINS, HOMA-IR, TC, TG, LDL-c, and hs-CRP were significantly higher in NGT-OB subgroup than in NGT-NW subgroup, while HDL-c were higher in NGT-NW subgroup than in NGT-OB subgroup (*P* < 0.05 or *P* < 0.01). Compared to NGT-OB subgroup, the levels of SBP, DBP, FPG, 2hPG, HbA1c, FINS, HOMA-IR, IL-6, hs-CRP, and TG were significantly increased in T2DM-OB subgroup, while the HOMA-*β* and HDL-c levels were decreased in T2DM-OB subgroup (*P* < 0.05 or *P* < 0.01). Compared to T2DM-NW subgroup, the levels of BMI, waist circumferences, WHR, DBP, FINS, HOMA-IR, IL-6, hs-CRP, and TG were higher in T2DM-OB subgroup. The HDL-c and HOMA-*β* were higher in T2DM-NW subgroup than that in T2DM-OB subgroup (*P* < 0.05 or *P* < 0.01).

### 3.2. The Changes in Plasma Progranulin Concentrations in Obesity and Type 2 Diabetes

**Figure 1:**
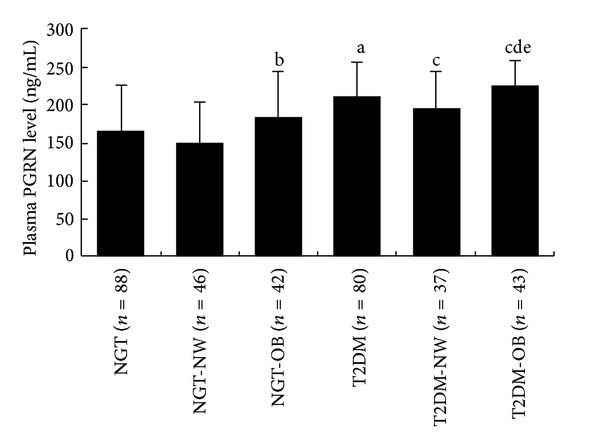
Plasma PGRN levels in different groups. Data are presented as means ± SD.  ^a^ *P* < 0.01 compared with NGT;  ^b^ *P* < 0.05 compared with NGT-NW;  ^c^ *P* < 0.01 compared with NGT-NW;  ^d^ *P* < 0.01 compared with NGT-OB;  ^e^ *P* < 0.05 compared with T2DM-NW.

There were no significant differences in plasma PGRN *concentrations* between men and women ((190.11 ± 56.63) versus (185.70 ± 59.54) ng/mL, *P* = 0.636). Compared to NGT group, T2DM group displayed a significant increase in the PGRN *concentrations* (165.44 ± 60.67 versus 211.52 ± 44.84 ng/mL, *P* < 0.01, Figure 1). Plasma PGRN *concentrations* in four subgroups are the followings: T2DM-OB subgroup (225.22 ± 34.39 ng/mL), T2DM-NW subgroup (195.59 ± 50.47 ng/mL), NGT-OB subgroup (183.79 ± 61.63 ng/mL), and NGT-NW subgroup (148.69 ± 55.27 ng/mL). T2DM-OB subgroup had significant higher levels of PGRN than those in T2DM-NW subgroup (*P* < 0.05) and in NGT-OB subgroup (*P* < 0.01).

**Figure 2:**
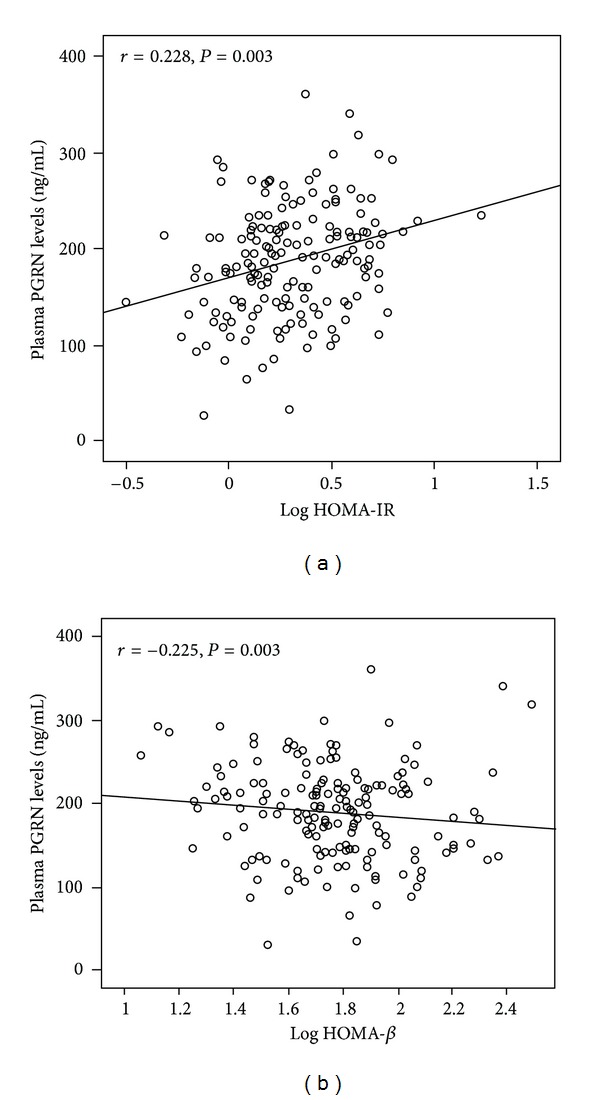
Scatter plots showing the correlation of plasma PGRN levels with HOMA-IR and HOMA-*β* in all subjects. (a) The plasma PGRN levels positively correlated with HOMA-IR. (b) The plasma PGRN levels negatively correlated with HOMA-*β*.

Bivariate correlation analysis revealed that the plasma PGRN *concentrations* correlated positively and significantly with weight, waist circumferences, HOMA-IR (Figure 2(a)) and FINS (*r* = 0.175, 0.191, 0.228, 0.172, resp., *P* < 0.05, Table 2). The PGRN *concentrations* also correlated positively and significantly with BMI, SBP, FPG, 2hPG, HbA1C, TG, and IL-6 significantly (*r* = 0.286, 0.256, 0.348, 0.292, 0.276, 0.221, 0.383, resp., *P* < 0.01, Table 2), while correlating negatively and significantly with HOMA-*β*  (*r* = −0.225, *P* < 0.05, Figure 2(b)). Multivariable linear regression models revealed that BMI, HbA1c, IL-6, and TG were independently related with the plasma PGRN levels (*β* = 0.191, 0.473, 0.181, 0.151, resp., *P* < 0.05). The multiple regression equation was as follows: *Y*_PGRN_ = −57.828 + 3.237*X*_BMI_ + 30.596*X*_HbA1c_  +  3.130*X*_IL-6_ + 8.722*X*_TG_.

## 4. Discussion

PGRN is a 68–88 kDa growth factor with seven and one-half granulin (GRN) motifs connected by short linker domains. It was originally discovered by Anakwe and Gerton in 1990 [14]. PGRN catches our attention because it is a secreted protein with inflammatory properties and was recently identified as a novel adipokine. In addition, the changes of circulating PGRN brought about by obesity and type 2 diabetes, respectively, in Chinese subjects are still unclear.

In our study, we found that plasma PGRN concentrations were 1.3-fold (*P* < 0.01) higher in normal-weight type 2 diabetes patients, compared to those of healthy subjects. The PGRN concentrations were lower than those in previous study which conducted by Youn et al. in the Korean population, and this may be because our subjects have lower BMI which may contribute to the increase of PGRN concentrations in type 2 diabetes patients. In addition, the most recent study reported that renal elimination is a major route for circulation PGRN [15]. Therefore, we tested serum creatinine for all subjects and only recruited the population with normal kidney function. Correlation analysis showed that plasma PGRN levels were positively correlated to FPG and 2hPG. Multivariable linear regression models revealed that HbA1c was an independent predictor of plasma PGRN levels. These results indicate that type 2 diabetes could increase plasma PGRN concentrations independently, and the circulating PGRN concentrations could increase with deteriorating glucose metabolism.

Gene expression analysis in previous studies revealed that both isolated adipocytes and cells of the adipose tissue matrix express progranulin gene [8, 16]. Progranulin gene expression is significantly higher in visceral fat compared with subcutaneous fat [8]. Matsubara et al. [16] find that ablation of PGRN gene protects against HFD-induced obesity in mice. Moreover, recombinant mouse PGRN (rmPGRN) treatment shows a higher probability that mice will become obese when fed a HFD [16]. That research demonstrates a significant relationship between PGRN and obesity. In the present study, we found that obese patients with normal glucose tolerance have 1.2-fold (*P* < 0.05) higher plasma PGRN concentrations compared to healthy subjects. Furthermore, obese type 2 diabetes patients have 1.5-fold (*P* < 0.01) higher PGRN concentrations than healthy subjects. There was an indication that obesity could elevate the plasma PGRN concentrations independently, and both obesity and abnormal glucose tolerance contribute to the increase of plasma PGRN concentrations. In our study, we also found a significant positive correlation between PGRN and waist circumferences. Furthermore, we found that plasma PGRN concentrations positively correlated to LDL-c and TG concentrations and negatively correlated to HDL-c. Those findings suggested that PGRN is associated with central obesity and lipid metabolism disorders in Chinese subjects.

Both type 2 diabetes and obesity are associated with a state of chronic low-grade inflammation which is characterized by increased pro-inflammatory factors and decreased anti-inflammatory factors. Those pro-inflammatory factors increase the accumulation of macrophages in adipose tissue, while the increased macrocytes could stimulate the release of pro-inflammatory factors from adipose tissue. Youn and colleagues [8] found that PGRN promote macrophages infiltration into white adipose tissues by ERK pathway in vitro and induce inflammatory response in adipose tissue. A recent study demonstrates that rmPGRN could stimulate the adipocytes to release more IL-6 and the increased secretion of IL-6 by TNF-*α* was completely blocked by ablation of PGRN gene in 3T3-L1 adipose cells [16]. Those findings suggested that PGRN could promote inflammatory response by increasing the secretion of proinflammatory factors. In our study, we found that IL-6 levels were higher in patients with both diabetes and obesity than in those with diabetes and normal-weight and those with obesity and normal glucose tolerance. Moreover, the IL-6 levels were lowest in normal subjects. We also found that PGRN correlated positively with IL-6, which was an independent predictor of PGRN. Taking these together, the results suggested that concentrations of plasma PGRN and the extent of chronic inflammation are enhanced along with the aggravation of glycolipid metabolic disorders. 

Insulin resistance is a key feature of obesity and type 2 diabetes and can directly result in hyperinsulinemia. Recently, a report shows that PGRN could induce insulin resistance through stimulating IL-6 expression in adipocytes [16]. Meanwhile, many studies find that IL-6 could increase the expression of cytokine signaling-3 (SOCS3) via activation of JAK-STAT signaling pathway in adipocytes to inhibit tyrosine phosphorylation of insulin receptor substrate (IRS-1), leading to impaired insulin signaling [17–20]. In our research, we found that plasma PGRN levels correlated significantly and positively with HOMA-IR and FINS, while negatively correlating with HOMA-*β*. Those results indicated that circulating PGRN levels correlated significantly positively with insulin resistance and negatively correlated with pancreatic *β*-cell function. Combined with the aforementioned relationship between PGRN and IL-6 in our study, we speculated that one of the mechanisms responsible for PGRN-induced insulin resistance may be associated with increased IL-6 levels. However, other possible factors may play a role in this process as well and need to be investigated further. 

 As yet, there are some limitations in our study that require emphasis. First, the sample size is small, and the nonsignificant associations between PGRN and some factors become statistically significant if larger samples were studied. Second, our study is a cross-sectional research, and therefore causality of PGRN and IL-6 cannot be established. 

In conclusion, we demonstrated for the first time that plasma PGRN concentrations increased in Chinese patients with type 2 diabetes and obesity. The concentrations of plasma PGRN correlated closely with the glycolipid metabolic disorder, chronic inflammation, and insulin resistance, suggesting PGRN may contribute to the pathogenesis of insulin resistance in humans. Thus PGRN could be a potential therapeutic target for management of type 2 diabetes and obesity.

## Figures and Tables

**Table 1:** Anthropometric and metabolic characteristics of the study groups.

	NGT		T2DM	
	NGT-NW	NGT-OB	T2DM-NW	T2DM-OB
*N* (M/F)	46 (15/31)	42 (20/22)	37 (11/26)	43 (18/25)
Age (year)	58.54 ± 5.96	59.10 ± 7.43	61.65 ± 6.96	60.86 ± 7.22
Weight (kg)	55.83 ± 5.74	68.79 ± 8.04^b^	55.54 ± 6.27	69.30 ± 8.51^f^
BMI (kg/cm^2^)	22.36 ± 1.75	27.81 ± 3.10^b^	22.97 ± 1.47	27.78 ± 2.25^f^
WC (cm)	78.17 ± 5.72	91.69 ± 5.42^b^	80.38 ± 6.20	91.81 ± 5.43^f^
WHR	0.86 ± 0.04	0.92 ± 0.05^b^	0.87 ± 0.05	0.91 ± 0.04^f^
SBP (mm Hg)	118.41 ± 9.45	122.79 ± 9.61	133.46 ± 15.65^bd^	127.43 ± 14.45^bd^
DBP (mm Hg)	74.20 ± 6.99	74.67 ± 7.25	74.49 ± 10.09	75.52 ± 8.81^ace^
FPG (mmol/L)	5.25 ± 0.33	5.40 ± 0.43	7.28 ± 1.34^bd^	7.46 ± 1.13^bd^
2hPG (mmol/L)	6.28 ± 1.03	6.00 ± 1.14	13.58 ± 2.99^bd^	13.09 ± 3.30^bd^
FINS (mU/L)	5.61 ± 2.58	8.87 ± 4.76^a^	7.58 ± 3.88	12.27 ± 7.04^bdf^
HbA1c (%)	5.49 ± 0.32	5.57 ± 0.35	6.79 ± 0.78^bd^	6.79 ± 0.80^bd^
TC (mmol/L)	4.43 ± 0.91	4.82 ± 1.00^a^	4.59 ± 0.84	4.85 ± 0.92^a^
TG (mmol/L)	1.07 ± 0.51	1.48 ± 0.84^a^	1.50 ± 0.80^a^	2.04 ± 1.37^bce^
HDL-c (mmol/L)	1.43 ± 0.27	1.28 ± 0.32^a^	1.31 ± 0.35	1.14 ± 0.23^bce^
LDL-c (mmol/L)	2.47 ± 0.69	2.84 ± 0.78^a^	2.55 ± 0.64	2.82 ± 0.70^a^
PGRN (ng/mL)	148.69 ± 55.27	183.79 ± 61.63^a^	195.59 ± 50.47^b^	225.22 ± 34.39^bde^
IL-6 (pg/mL)	2.57 ± 1.78	3.73 ± 2.69	4.46 ± 3.37^a^	7.68 ± 3.14^bde^
hsCRP (mg/dL)	0.24 ± 0.13	0.35 ± 0.26^a^	0.32 ± 0.18^a^	0.43 ± 0.29^bdf^
HOMA-IR	1.32 ± 0.64	2.12 ± 1.14^a^	2.43 ± 1.22^b^	4.07 ± 2.49^bdf^
HOMA-*β*	93.42 ± 39.60	90.90 ± 44.27	63.51 ± 32.09^bd^	43.96 ± 26.08^bde^

Data are presented as means ± SD. NGT: normal glucose tolerance; T2DM: type 2 diabetes mellitus; NW: normal weight; OB: obesity; BMI: body mass index; WC: waist circumference; WHR: waist hip ratio; SBP: systolic blood pressure; DBP: diastolic blood pressure; FPG: fasting plasma glucose; 2hPG: 2h postchallenge plasma glucose; FINS: fasting serum insulin; HOMA-IR: Homeostasis Model Assessment for insulin resistance; HOMA-*β*: Homeostasis Model Assessment for beta-cell function; TC: total cholesterol; TG: triglyceride; HDL-c: high-density lipoprotein-cholesterol; LDL-c: low-density lipoprotein-cholesterol; hs-CRP: high-sensitivity C-reaction protein. ^a^ *P *< 0.05 compared with NGT-NW, ^b^ *P* < 0.01 compared with NGT-NW; ^c^ *P *< 0.05 compared with NGT-OB, ^d^ *P* < 0.01 compared with NGT-OB, ^e^ *P *< 0.05 compared with T2DM-NW, and ^f^ *P* < 0.01 compared with T2DM-NW.

**Table 2:** Univariate associations between plasma PGRN levels and metabolic parameters.

	*r *	*P* value
Weight (kg)	0.175	0.023
BMI (kg/m^2^)	0.286	<0.001
WC (cm)	0.191	0.013
WHR	0.042	0.589
SBP (mmHg)	0.256	0.001
DBP (mmHg)	0.138	0.074
FPG (mmol/L)	0.348	<0.001
2hPG (mmol/L)	0.292	<0.001
FINS (mU/L)	0.172	0.025
HbA1c (%)	0.276	<0.001
TC (mmol/L)	0.126	0.103
TG (mmol/L)	0.221	0.004
HDL-c (mmol/L)	−0.110	0.156
LDL-c (mmol/L)	0.059	0.450
IL-6 (pg/mL)	0.383	<0.001
HOMA-IR	0.228	0.003
HOMA-*β*	−0.225	0.003

WHR: waist hip ratio; SBP: systolic blood pressure; DBP: diastolic blood pressure; HOMA-IR: Homeostasis Model Assessment for insulin resistance; HOMA-*β*: Homeostasis Model Assessment for beta-cell function; TC: total cholesterol; TG: triglyceride; HDL-c: high-density lipoprotein-cholesterol; LDL-c: low-density lipoprotein-cholesterol.
